# Identification of 3 novel *VHL* germ-line mutations in Danish VHL patients

**DOI:** 10.1186/1471-2350-13-54

**Published:** 2012-07-16

**Authors:** Mette Dandanell, Lennart Friis-Hansen, Lone Sunde, Finn C Nielsen, Thomas v O Hansen

**Affiliations:** 1 Center for Genomic Medicine, Rigshospitalet, Copenhagen University Hospital, Copenhagen, Denmark; 2 Department of Clinical Genetics, Aarhus University Hospital, Aarhus, Denmark; 3 Department of Clinical Genetics, Aalborg Hospital, Aalborg, Denmark

**Keywords:** von Hippel-Lindau disease, VHL, Germ-line mutations, Danish population

## Abstract

**Background:**

von Hippel-Lindau (VHL) disease is a hereditary cancer syndrome in which the patients develop retinal and central nervous system hemangioblastomas, pheochromocytomas and clear-cell renal tumors. The autosomal dominant disease is caused by mutations in the *VHL* gene.

**Methods:**

*VHL* mutational analysis was carried out by sequencing of the coding sequence and by multiplex ligation-dependent probe amplification analysis. The functional consequence of the variants was investigated using *in silico* prediction tools.

**Results:**

A total of 289 probands suspected of having VHL syndrome have been screened for mutations in the *VHL* gene. Twenty-six different *VHL* mutations were identified in 36 families including one in-frame duplication, two frame-shift mutations, four nonsense mutations, twelve missense mutations, three intronic mutations and four large genomic rearrangements. Three of these mutations (c.319 C > T, c.342_343dupGGT and c.520_521dupAA) were novel.

**Conclusions:**

In this study we report the *VHL* germ-line mutations found in Danish families. We found three novel *VHL* mutations where two were classified as pathogenic and the latter was classified as a variant of unknown significance. Together, our findings contribute to the interpretation of the potential pathogenicity of *VHL* germ-line mutations.

## Background

**Figure 1:**
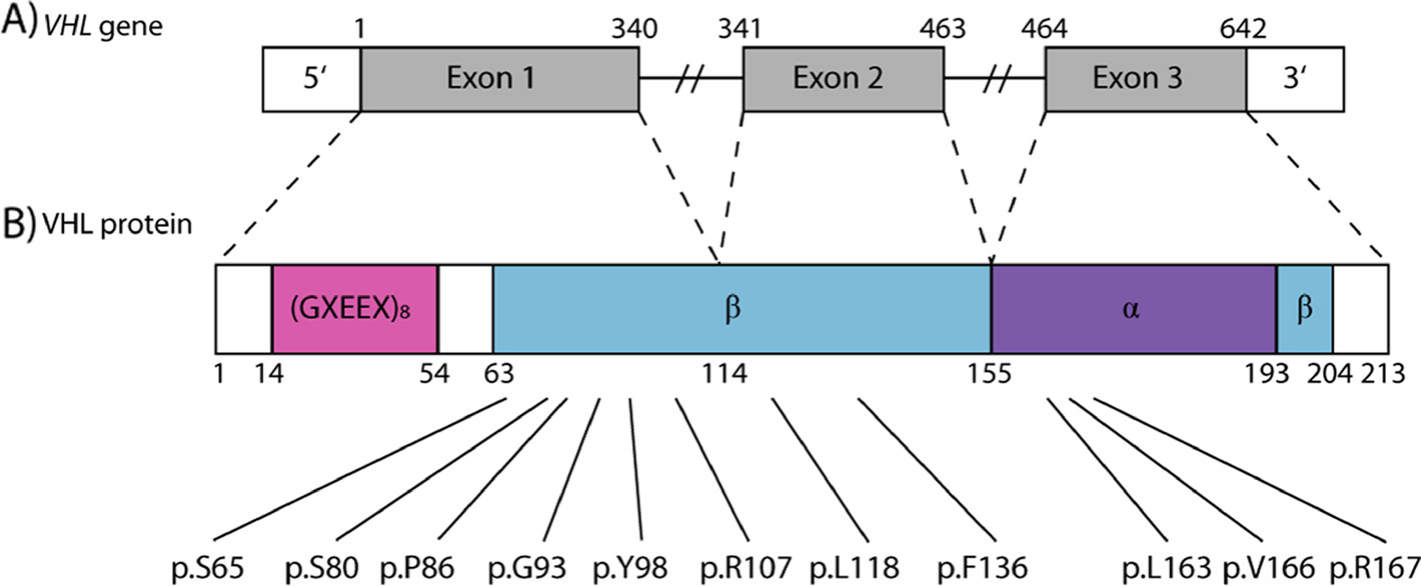
**von Hippel-Lindau gene and protein structure. A**) The *VHL* gene comprises three exons (gray). Nucleotide number is indicated above the gene structure. **B**) The VHL protein structure with (GXEEX)_8_ repeat motif (pink), α-domain (purple) and β-domain (blue). Codon number is indicated below the protein structure. The eleven residues subjected for missense mutations are indicated below the figure.

von Hippel-Lindau (VHL) disease (OMIM 193300) is a dominantly inherited cancer syndrome characterized by central nervous system and retinal hemangioblastomas, clear cell renal carcinoma, neuroendocrine tumors and cysts of the pancreas, pheochromocytomas, endolymphatic sac tumors and/or papillary cystadenomas of the epididymis and broad ligament [1]. Patients without pheochromocytomas are classified as VHL type 1 and those with pheochromocytoma as VHL type 2. Type 2 patients are further divided into three subgroups: type 2A patients with low risk of renal cell carcinomas, type 2B patients with high risk of renal cell carcinomas and type 2 C patients with isolated pheochromocytoma [2].

von Hippel-Lindau disease is caused by germ-line mutations in the *VHL* tumor suppressor gene located on the short arm of chromosome 3 (3p25-26). The gene spans 10 kb, is composed of three exons (Figure 1) and encodes two proteins (pVHL19 and pVHL30) [3-5]. The complete VHL protein consists of 214 amino acids and has two structural domains: the α-domain and the β-domain [6].

The best characterized function of pVHLs is its role as a substrate recognition component of the E3 ubiquitin protein ligase complex comprising pVHL, Elongin C, Elongin B, Cullin 2 and Rbx1. In this complex, pVHL targets the α-subunit of hypoxia inducible factor 1 (HIF-1α) and hypoxia inducible factor 2 (HIF-2α, also known as EPAS1) transcription factors for ubiquitin-mediated proteolysis. The HIF transcription factors are involved in coordination of the cellular response to hypoxia and in transcriptional regulation of hypoxia inducible genes, including *VEGFPDGFTGFα* and *EPO*. pVHL inactivation leads to stabilization of HIF-1 and HIF-2 and subsequent transcriptional activation of HIF-induced target genes [2].

Since 1998 we have performed the screening of Danish patients suspected of having VHL disease. The clinical characterization of the Danish *VHL* mutation carriers has recently been published [7,8] and in this study we report the *VHL* germ-line mutations identified during this period.

## Methods

### Patients

The national Danish guidelines recommend mutational *VHL* screening when a patient 1) exhibits at least two of the following VHL manifestations: familial history of renal cancers, paragangliomas, hemangioblastomas, endolymphatic sac tumors, neuroendocrine tumors, cysts of the pancreas and/or pheochromocytomas 2) has one VHL affected first degree relative and exhibits at least one of the mentioned manifestations [9]. Following verbal and written consent blood samples were collected from the probands. All family relatives are offered pre-symptomatic testing and genetic counseling.

### VHL screening

Genomic DNA was purified from whole blood using either the QIAamp DNA mini kit (Qiagen, Hilden, Germany) or Maxwell 16 blood DNA purification kit (Promega, Stockholm, Sweden) according to manufacturer’s instructions. The *VHL* exons were amplified using intronic primer pairs flanking each exon (Table 1) and the PCR products were subsequently sequenced using an ABI3730 DNA analyzer (Applied Biosystems, Foster City, CA). Moreover, genomic DNA was examined for large genomic rearrangements by multiplex ligation-dependant probe amplification (MLPA) analysis using the salsa MLPA P016 *VHL* kit (MRC-Holland, Amsterdam, Holland). All sequence variants were verified in an independent blood sample. *VHL* variants are numbered according to accession number [GenBank: NM_000551] following the guidelines from the Human Genetic Variation Society (http://www.hgvs.org/mutnomen).

**Table 1:** **Forward and reverse primers used for PCR amplification of the *VHL* gene**

	**Forward Primer**	**Reverse Primer**
Exon 1	5’-gcgcgcgaagactacggaggt-3’	5’-gaatgctctgacgcttac-3’
Exon 2	5’-gtggctctttaacaacctttgct-3’	5’-cctgtacttaccacaacaaccttatc-3’
Exon 3	5’-gtggaccctagtctgtcactgagg-3’	5’-agactcatcagtaccatcaaaag-3’

### In silico analysis

*In silico* prediction of the functional consequence of the missense variants was performed using the web-based program Align GVGD [10]. Align GVGD grades of > = C25 were considered likely deleterious. The following three splice site prediction programs were used to predict the variants influence on splicing pattern: http://www.fruitfly.org/seq_tools/splice.html[11], http://www.cbs.dtu.dk/services/NetGene2[12] and http://www.umd.be/SSF[13]. The genomic sequence spanning the intron mutations was submitted according to the guidelines of each program and default settings were used in all predictions.

## Results and discussion

Until August 2011, 289 Danish patients had been screened for mutations in the *VHL* gene by direct sequencing and MLPA analysis. Germ-line mutations were detected in 36 families and the mutations were distributed widely throughout the gene except for the first ~60 amino acids (Figure 1). The pathogenicity of the 26 different mutations found in the *VHL* gene were classified using published data and *in silico* prediction analysis (Table 2).

**Table 2:** ** *VHL* mutations identified in Danish patients with VHL suspected disease**

**Family ID**	**Exon**	**Nucleotide**	**Protein**	** *In silico* **	**Interpretation**	**References**
04012		c.1-?_340 + ?del			Pathogenic	[14,15]
04013						
04231						
00117		c.1-?_463 + ?del			Pathogenic	[14]
04178						
00108	1	c.194 C > T	p.Ser65Leu	C65	Pathogenic	[14,16]
00118	1	c.194 C > G	p.Ser65Trp	C65	Pathogenic	[14-17]
04025						
00116	1	c.239 G > T	p.Ser80Ile	C15	Pathogenic	[14-19]
04268	1	c.257 C > T	p.Pro86Leu	C0	Pathogenic	[15-17]
00101	1	c.278 G > A	p.Gly93Asp	C0	Pathogenic	[14,16]
00113	1	c.293A > G	p.Tyr98Cys	C65	Pathogenic	[15,20]
00107	1	c.319 C > T	p.Arg107Cys	C25	Pathogenic	Novel
00109	1	c.337 C > T	p.Arg113X		Pathogenic	[21]
04283		c.340 + 5 G > C			Polymorphism	[17,22,23]
00121	1	c.342_343dupGGT	p.Gly114_His115insGly		Unknown	Novel
00103		c.341-?_463 + ?del			Pathogenic	[14,18]
00105						
04208						
04078						
00107	2	c.353 T > C	p.Leu118Pro	C25	Pathogenic	[16,17,24]
00102	2	c.407 T > C	p.Phe136Ser	C55	Pathogenic	[16,17]
00104	2	c.433 C > T	p.Gln145X		Pathogenic	[17]
04008		c.463 + 1 G > T			Pathogenic	[16,18]
04030		c.463 + 8 C > T			Unknown	[25]
04163						
04135		c.464-?_642 + ?del			Pathogenic	[14,15]
04168						
04181						
04237						
00121	3	c.481 C > T	p.Arg161X		Pathogenic	[14,16,18,22]
04015	3	c.488 T > A	p.Leu163His	C0	Pathogenic	[22]
00123	3	c.496 G > T	p.Val166Phe	C0	Pathogenic	[14,16,17]
00106	3	c.499 C > T	p.Arg167Trp	C65	Pathogenic	[16-18,24]
00112	3	c.520_521dupAA	p.Asn174LysfsX29		Pathogenic	Novel
04026	3	c.548 C > A	p.Ser183X		Pathogenic	[14,16]
04024	3	c.606dupA	p.Gln203ThrfsX53		Pathogenic	[17]

*In silico* predictions was performed using the web-based program Align GVGD and grades above 25 was considered likely deleterious.

### In-frame deletion, frame-shift and nonsense mutations

The novel c.342_343dupGGT mutation is an in-frame duplication inserting a glycine residue between glycine-114 and histidine-115 (p.Gly114_His115insGly) in the VHL protein. The GGT duplication is located in the 5’ end of exon 2 close to the splice acceptor site. However, *in silico* analysis did not predict any major changes in the splicing pattern and thus the pathogenicity of the p.Gly114_His115insGly mutation remains unknown. Since only the proband was screened it has not been possible to examine if this specific variant co-segregates with VHL disease.

The two frame-shift mutations, c.520_521dupAA (p.Asn174LysfsX28) and c.606dupA (p.Gln203ThrfsX55), both located in exon 3, introduce two new termination codons. The c.520_521dupAA frame-shift leads to a truncated pVHL, whereas the c.606dupA frame-shift results in a larger pVHL. To our knowledge only the c.606dupA mutations has been described before [17], however both mutations are classified as pathogenic.

The four nonsense mutations found in exon 1 (p.Arg113X), exon 2 (p.Gln145X) and exon 3 (p.Arg161X and p.Ser163X) have previously been reported and are all classified as pathogenic [14,16-18,21,22].

### Missense mutations

Seven of the twelve identified missense mutations were located in exon 1, two in exon 2 and three in exon 3 (Figure 1). Besides these, a known polymorphism (c.74 C > T, p.Pro25Leu) [19,26] was found in six unrelated Danish families.

Two mutations changed the serine-65 residue to leucine (p.Ser65Leu) and tryptophan (p.Ser65Trp), respectively. The p.Ser65Leu mutation was found in several members of the same family; four mutation- or obligate carriers were diagnosed with VHL disease between 27 and 46 years, five carriers were unaffected (0–22 years), two obligate carriers died unaffected (85 and 88 years) and the parents of three obligate carriers died unaffected (70 and 92 years). The p.Ser65Trp mutation was found in two unrelated individuals with VHL related disease. Serine-65 mutations are known to disrupt binding of pVHL to HIF-1α [27] and are observed in several type 1 VHL affected families [14,15,17]. *In silico* analysis predicted a pathogenic effect (class C65) of both the p.Ser65Leu and the p.Ser65Trp mutations supporting the hypothesis that both mutations are pathogenic. The observation of unaffected mutation carriers may indicate reduced penetrance of the p.Ser65Leu mutation or the co-inheritance of a protective genetic modifier in some family members.

The p.Ser80Ile mutation was found in a single patient. Although *in silico* analysis classify the mutation of low clinical significance (class C15) the mutation is expected to disrupt the three-dimensional structure of pVHL [19] indicating that the mutation is pathogenic. The mutation has previously been identified in a Hungarian type 2 VHL affected family where all members except the proband’s mother showed clinical manifestations of VHL disease [19].

The p.Pro86Leu mutation was found in one individual and the p.Gly93Asp mutation was found in eight members of one family. The mutations have previously been associated with type 1 and type 2 VHL disease, respectively [14,16,17]. The two mutations are both located in the β-domain of pVHL (Figure 1) where the proline-86 residue is important for the structural integrity of the β sandwich [6]. Moreover, the p.Pro86Leu mutation was reported as a *VHL* hotspot [6,28]. These findings indicate a pathogenic role of the two missense mutations regardless of the result of the *in silico* analysis that predicted the mutations to be without clinical significance (class 0).

The p.Tyr98Cys missense mutation was found in two patients from the same family and has previously been associated with type 2 VHL disease [15]. Another germ-line mutation at tyrosine-98 (p.Tyr98His) was reported as a German founder mutation [20]. The tyrosine-98 residue is located on the surface of the β-sandwich [6] and *in silico* analysis predicted the mutation to be of high clinical significance (class C65).

Both a mother and her son were found to carry two *VHL* missense mutations (p.Arg107Cys and p.Leu118Pro) proving that both mutations are present on the same allele. Changes of the arginine-107 residue to glycine and histidine have previously been reported [18,29] whereas the p.Arg107Cys missense mutation is novel. The p.Leu118Pro mutation has been associated with type 1 VHL disease [24]. Hence, clinical findings and Align GVGD classifications (class C25) indicates a pathogenic role of both mutations although the contribution of each mutation to the phenotype is unknown.

The p.Phe136Ser mutation was found in a single patient. This mutation has previous been associated with type 1 VHL disease [16,17] and together with *in silico* analysis (class C55) this suggest that the mutation is pathogenic.

The p.Leu163His, p.Val166Phe and p.Arg167Trp mutations are all located in the α-domain of pVHL (Figure 1). *In silico* analysis does not indicate a pathogenic effect of p.Leu163His and p.Val166Phe (class C0). However, Leucine-163 and valine-166 are contact residues when pVHL binds to elongin C [30] and mutations at the leucine-163 residue have shown to impair the ability of pVHL to target HIF-1α for destruction [31]. These findings suggest that both mutations are pathogenic. Arginine-167 has a structural role in stabilizing the H1 helix and the α-β domain interface and was previously reported as the most frequently mutated residue in *VHL*[6]. The importance of arginine-167 is supported by *in silico* analysis classifying the p.Arg167Trp mutation as pathogenic (class C65).

### Intronic mutations

We identified three intronic mutations located in intron 1 (c.340 + 5 G > C) and intron 2 (c.463 + 1 G > T and c.463 + 8 C > T). The c. 340 + 5 G > C intronic mutation was previously observed in a VHL family *in trans* with a pathogenic *VHL* mutation [22,23] suggesting it is a benign polymorphism. This is supported by *in silico* analysis showing no major changes in splicing pattern. The c.463 + 1 G > T intronic mutation disrupt the highly conserved splice donor site [32] and *in silico* analysis predicted skipping of *VHL* exon 2. Combined with an earlier described association with type 1 VHL disease [18] this clearly suggests a pathogenic effect of this mutation. The c.463 + 8 C > T mutation was found in two unrelated individuals. The mutation was previously described as a variant of unknown significance [25]. *In silico* analysis suggested no major changes in the splicing pattern indicating that the variant might be a benign polymorphism. However, functional studies or analysis of co-segregation are needed to confirm this.

### Large genomic rearrangements

Large genomic rearrangements were identified in thirteen Danish families. Deletion of exon 1 (c.1-?_340 + ?del) was found in three families, deletion of both exon 1 and 2 (c.1-?_463 + ?del) was found in two families whereas deletion of either exon 2 (c.341-?_463 + ?del) or exon 3 (c.464-?_642 + ?del) was found in four families each. Although the exact break point of the four different deletions is unknown similar deletions have previously been reported as pathogenic [14,15,18]. Large genomic rearrangements leading to truncated versions of pVHL are primarily associated with type 1 VHL disease [33].

Altogether, we found 33.4% missense mutations, 11.1% nonsense mutations, 11.1% intronic mutations, 2.7% in-frame mutations, 5.6% frame-shift mutations and 36.1% large genomic rearrangement. This spectrum of mutations is in concordance with a recently published study [34], although we found less missense mutations (33.4% vs 52%) and a higher prevalence of large genomic rearrangements (36.1% vs 11%).

## Conclusions

In conclusion, we have screened 289 Danish probands with VHL suspected disease and identified 26 mutations in 36 families. We classified all large genomic rearrangements and all nonsense- and missense mutations as pathogenic. The two frame-shift mutations were classified as pathogenic, whereas the in-frame duplication was classified as a variant of unknown significance. Of the three intron variants found, one was a benign polymorphism, one was pathogenic and one was a variant of unknown significance. Of these mutations the missense mutation c.319 C > T (p.Arg107Cys), the in-frame duplication c.342_343dupGGT (p.GlyHis_His115insGly) and the frame-shift mutation c.520_521dupAA (p.Asn174LysfsX29) were found to be novel.

## Competing interests

The authors declare that they have no competing interests.

## Authors’ contributions

TvOH and LFH designed the study and LS was involved in recruitment of patients. MD and TvOH performed the *in silico* analyses. MD, TvOH, FCN, LS and LFH have been involved in drafting the manuscript or revising it critically for important intellectual content. All authors read and approved the final version of the manuscript.

## Pre-publication history

The pre-publication history for this paper can be accessed here:

http://www.biomedcentral.com/1471-2350/13/54/prepub
